# Protein Abundance of Drug Metabolizing Enzymes in Human Hepatitis C Livers

**DOI:** 10.3390/ijms24054543

**Published:** 2023-02-25

**Authors:** Marek Drozdzik, Joanna Lapczuk-Romanska, Christoph Wenzel, Lukasz Skalski, Sylwia Szeląg-Pieniek, Mariola Post, Arkadiusz Parus, Marta Syczewska, Mateusz Kurzawski, Stefan Oswald

**Affiliations:** 1 Department of Experimental and Clinical Pharmacology, Pomeranian Medical University, 70-111 Szczecin, Poland; 2 Department of Pharmacology, Center of Drug Absorption and Transport, University Medicine Greifswald, 17489 Greifswald, Germany; 3 Department of General and Transplantation Surgery, County Hospital, 71-455 Szczecin, Poland; 4 Department of Mechatronics, West Pomeranian University of Technology, 70-310 Szczecin, Poland; 5 Department of Infectious Diseases, Hepatology and Liver Transplantation, Pomeranian Medical University, 71-455 Szczecin, Poland; 6 Laboratory of Pharmacodynamics, Pomeranian Medical University, 71-899 Szczecin, Poland; 7 Institute of Pharmacology and Toxicology, Rostock University Medical Center, 18057 Rostock, Germany

**Keywords:** hepatitis C, liver, drug metabolizing enzymes

## Abstract

Hepatic drug metabolizing enzymes (DMEs), whose activity may be affected by liver diseases, are major determinants of drug pharmacokinetics. Hepatitis C liver samples in different functional states, i.e., the Child–Pugh class A (*n* = 30), B (*n* = 21) and C (*n* = 7) were analyzed for protein abundances (LC-MS/MS) and mRNA levels (qRT-PCR) of 9 CYPs and 4 UGTs enzymes. The protein levels of CYP1A1, CYP2B6, CYP2C8, CYP2C9, and CYP2D6 were not affected by the disease. In the Child–Pugh class A livers, a significant up-regulation of UGT1A1 (to 163% of the controls) was observed. The Child–Pugh class B was associated with down-regulation of the protein abundance of CYP2C19 (to 38% of the controls), CYP2E1 (to 54%), CYP3A4 (to 33%), UGT1A3 (to 69%), and UGT2B7 (to 56%). In the Child–Pugh class C livers, CYP1A2 was found to be reduced (to 52%). A significant trend in down-regulation of the protein abundance was documented for CYP1A2, CYP2C9, CYP3A4, CYP2E1, UGT2B7, and UGT2B15. The results of the study demonstrate that DMEs protein abundances in the liver are affected by hepatitis C virus infection and depend on the severity of the disease.

## 1. Introduction

Hepatitis C virus (HCV) is a small, enveloped RNA virus targeting hepatocytes, and its local replication and immune responses lead to liver damage, ultimately resulting in cirrhosis and/or hepatocellular carcinoma. The implementation of new treatment modalities, i.e., direct acting antiviral drugs (DAAs) have markedly improved therapeutic outcomes. However, the treatment success rate is determined by the HCV genotype, and its high rate and the erroneous nature of its viral replication result in a high prevalence of resistance-associated substitutions (with and without drug pressure) that may impact the efficacy of pharmacotherapy [1].

The currently available DAAs are subjected to metabolism via enzymes located mainly in the intestine and liver [2]. DAAs are substrates of cytochrome P450 (CYP)1A2—pibrentasvir, CYP2B6—velpatasvir, CYP2C8—dasabuvir, velpatasvir, CYP2C19—simeprevir, CYP3A4/5—glecaprevir, grazoprevir, voxilaprevir, daclatasvir, elbasvir, pibrentasvir, simeprevir, velpatasvir, and uridine 5′-diphospho-glucuronosyltransferase (UGT)1A1—pibrentasvir. Apart from being enzymatic substrates, some of the drugs produce inhibitory activity against enzymes, which complicates the enzyme/substrate/inhibitor interplay. The following are the enzymatic inhibitors: CYP1A2—glecaprevir, pibrentasvir; CYP3A4/5—asunaprevir, daclatasvir, dasabuvir, elbasvir, glecaprevir, grazoprevir, paritaprevir, pibrentasvir, simeprevir, velpatasvir; and UGT1A1—glecaprevir, and pibrentasvir [3,4].

The summaries of product information of some DAAs define that safety and efficacy have not been studied in HCV-infected patients with moderate or severe hepatic impairment (Child–Pugh score B or C) due to ethical concerns and methodological challenges. However, the European Medicines Agency (EMA) and the United States of America Food and Drug Administration (FDA) regulations recommend pharmacokinetic studies in patients with impaired hepatic function when it is likely that liver dysfunction may significantly affect pharmacokinetics (especially metabolism and biliary excretion) and dose adjustments might be needed [5,6].

Therefore, quantitative information about drug metabolizing enzymes (DMEs) levels in HCV livers is of clinical relevance. In this regard, physiologically based pharmacokinetic (PBPK) modeling and simulation may enable stratification of potential risks derived from the altered pharmacokinetics of administered drugs, prediction of oral drug bioavailability, and drug–drug interactions (DDIs). The findings of this study can be applied not only to agents targeting HCV, but also to other drugs (DMEs substrates) that are administered to patients with hepatitis C. This study provides information about DMEs proteomic data from only one pathological state of the liver stratified according to the Child–Pugh classification, and includes the largest number of cases published so far.

Our preliminary study revealed the impact of liver diseases on DMEs protein levels, and it included 21 cases of HCV livers (also used in the current analysis). The findings revealed that the disease entails significant decrease in CYP2E1, CYP3A5, and UGT2B7 protein abundance [7]. However, due to the limited number of cases, we could not analyze the data according to liver dysfunction stage. This study suggested that the protein abundance of DMEs was affected by both the type of liver pathology (hepatitis C, alcoholic liver disease, autoimmune hepatitis, primary biliary cholangitis, and primary sclerosing cholangitis) as well as the organ functional status (according to the Child–Pugh classification); however, in the latter analysis, samples with different liver pathologies were merged. The combined analysis of all liver pathologies suggested that the worsening of liver functions was associated with a significant decrease in protein abundance of CYP2E1 in class A, downregulation of CYP3A4 and UGT2B7 starting from class B, and CYP1A2, CYP2C8, and CYP2C9 appeared to be reduced in the Child–Pugh score C livers [7].

In the present study, we were able to substantially expand the number of HCV samples, which not only allowed us to provide quantitative proteomic (LC–MS/MS) data on DMEs status in HCV livers, but also to stratify DMEs expression levels according to the Child–Pugh classification, i.e., functional state of the liver. The information presented in this study is complementary to the findings on drug transporters in the liver in patients suffering from HCV infection, as the same subjects were included in the analysis [8].

## 2. Results

### 2.1. mRNA Expression

**Figure 1:**
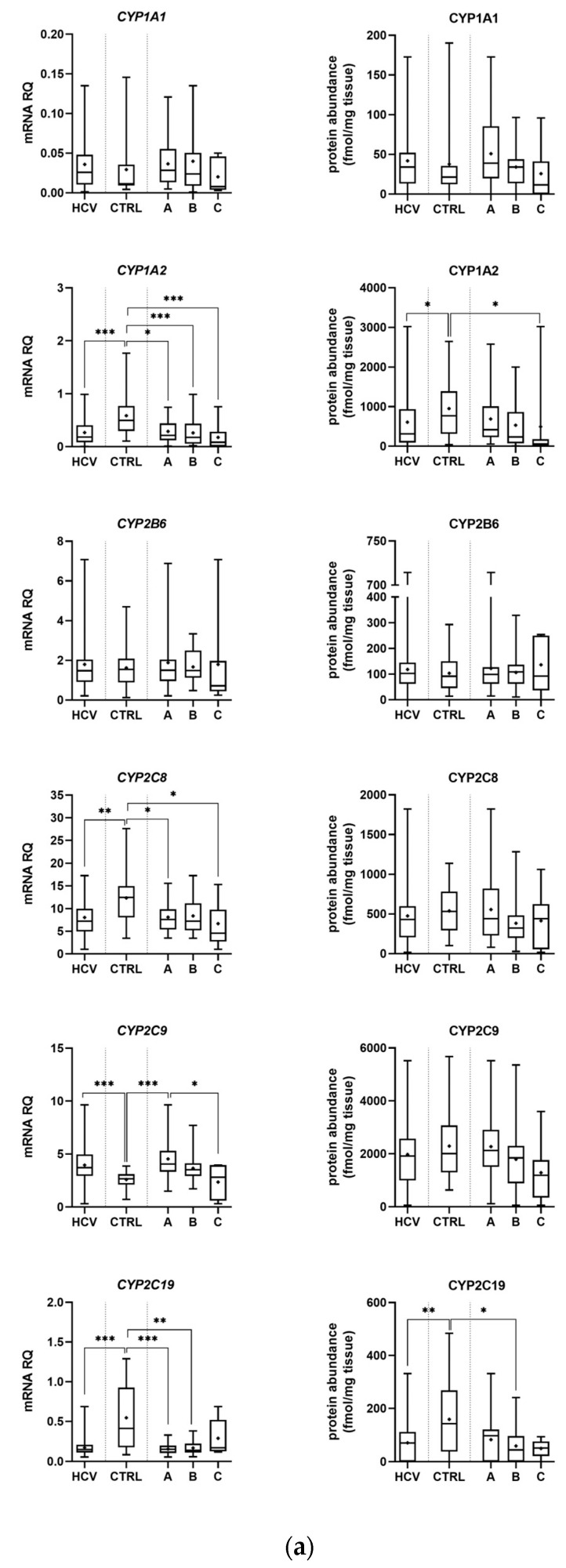

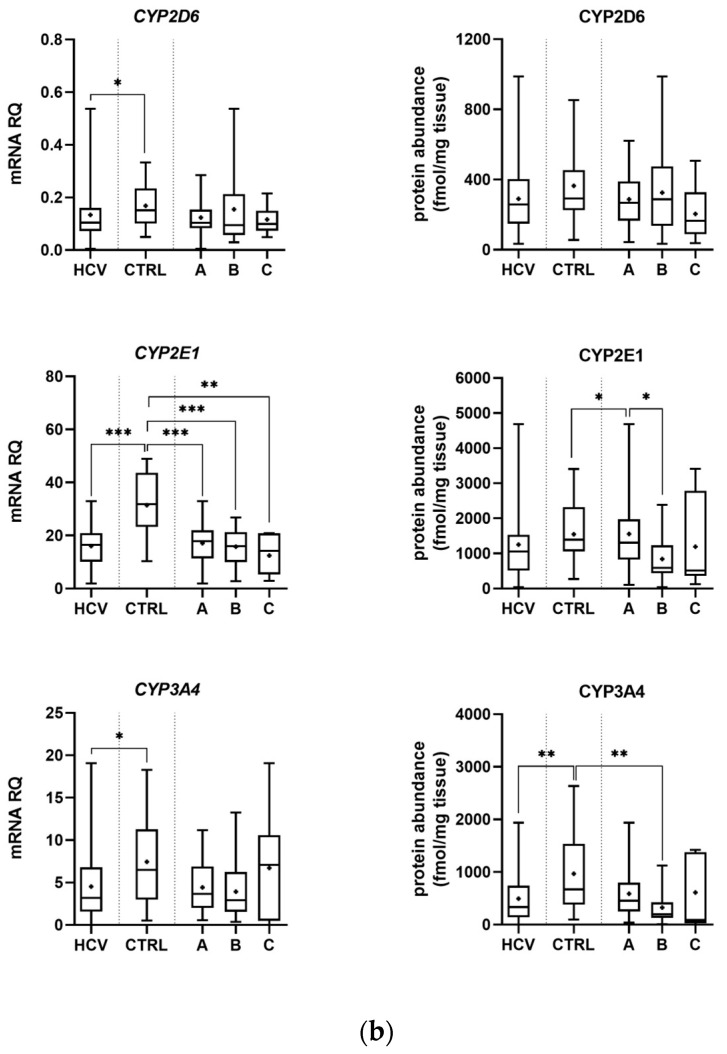
Gene expression (**left**) and protein abundance (**right**) of CYPs in hepatitis C (HCV, *n* = 58) liver tissues stratified according to the Child–Pugh score into stages: A (*n* = 30), B (*n* = 21), and C (*n* = 7), and the controls (CTRL, *n* = 20). The data are represented as box-plots of the median (horizontal line), 75th (top of box) and 25th (bottom of box) quartiles, the smallest and largest values (whiskers) and mean (+). mRNA levels of the analyzed genes are expressed as relative amounts to the mean of five housekeeping genes (*GAPDH, HMBS, PPIA, RPLP0, RPS9*). Statistically significant differences: * *p* < 0.05, ** *p* < 0.01, *** *p* < 0.001 (Wilcoxon-signed rank test in comparison to the controls). (**a**) Results for CYP1A1, 1A2, 2B6, 2C8, 2C9 and 2C19. (**b**) Results for CYP2D6, 2E1 and 3A4.

**Figure 2:**
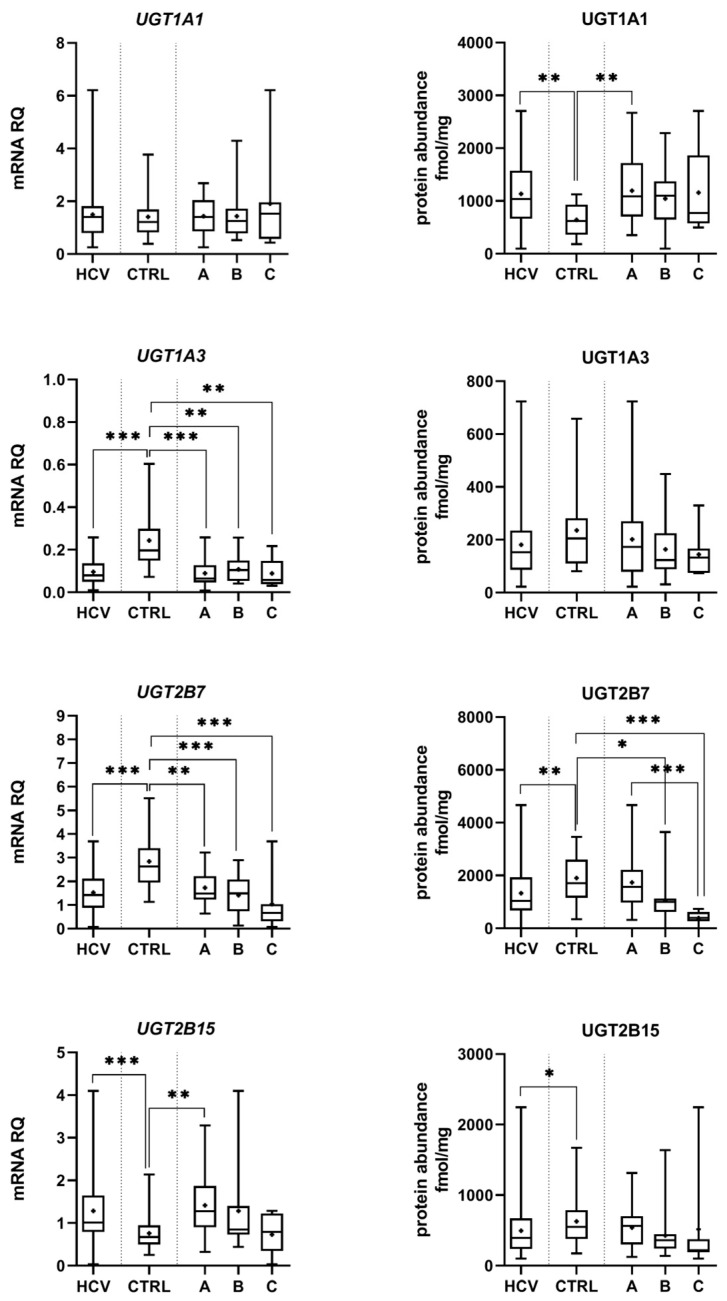
Gene expression (**left**) and protein abundance (**right**) of UGTs in hepatitis C (HCV, *n* = 58) liver tissues stratified according to the Child–Pugh score into stages: A (*n* = 30), B (*n* = 21), and C (*n* = 7), and the controls (CTRL, *n* = 20). The data are represented as box-plots of the median (horizontal line), 75th (top of box) and 25th (bottom of box) quartiles, the smallest and largest values (whiskers) and mean (+). mRNA levels of the analyzed genes are expressed as relative amounts to the mean of five housekeeping genes (*GAPDH, HMBS, PPIA, RPLP0, RPS9*). Statistically significant differences: * *p* < 0.05, ** *p* < 0.01, *** *p* < 0.001 (Wilcoxon-signed rank test in comparison to the controls).

In the control samples, a strong correlation (r_s_ > 0.6) between DMEs mRNA expression and protein abundance was observed. Only CYP2E1/CYP2E1 and CYP2C19/CYP2C19 did not demonstrate significant correlations (Table 1). However, HCV infection affected correlations between DMEs gene expression and protein levels, i.e., loss of strong correlation was seen in most enzymes, but in general the same trend in mRNA and protein abundance changes was observed (Table 1, Figure 1 and Figure 2, Supplementary Tables S1 and S2).

### 2.2. Protein Abundance

Protein abundance levels of several enzymes were not significantly affected by HCV infection, i.e., CYP1A1, CYP2B6, CYP2C8, CYP2C9, CYP2D6, and CYP3A5. The Child–Pugh class A livers were characterized by significant up-regulation of UGT1A1 (to 163% of the controls). The Child–Pugh class B was associated with down-regulation of CYP2C19 (to 38% of the controls), CYP2E1 (to 54% of the controls), CYP3A4 (to 33% of the controls), UGT1A3 (to 69% of the controls), and UGT2B7 (to 56% of the controls). Significant reductions in CYP1A2 (to 52% of the controls) and UGT2B7 (to 20% of the controls) in the Child–Pugh stage C were also noted (Figure 1 and Figure 2, Supplementary Table S2). The significant trend in the downregulation of CYP1A2, CYP2C9, CYP3A4, CYP2E1, UGT2B7, and UGT2B15 was documented.

The merged results of all HCV samples revealed that the disease did not significantly affect the protein levels of CYP1A1, CYP2B6, CYP2C8, CYP2C9, CYP2D6, CYP2E1, and UGT1A3 as compared to the control samples. Significant decrease in CYP1A2 (to 64% of the controls), CYP2C19 (to 45% of the controls), CYP3A4 (to 47% of the controls), and CYP3A5 (to 55% of the controls) as well as UGT2B7 (to 70% of the controls), UGT2B15 (to 79% of the controls), and marked increase in UGT1A1 (to 177% of the controls) abundances, were noted (Figure 1 and Figure 2, Supplementary Table S2).

**Figure 3:**
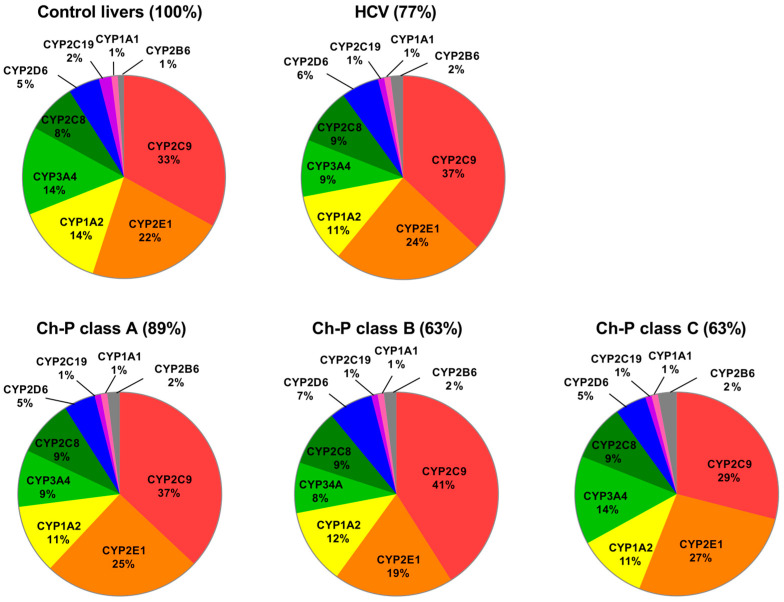
Pie charts of the individual enzyme protein amounts in HCV livers stratified according to the Child–Pugh score (A, B, and C class). The pie charts show the abundance of each enzyme as a percentage of the sum of all enzyme protein abundances. Percentages indicate a total enzyme protein abundance in comparison to the control livers (indicated as 100%).

The percentage contributions of all investigated CYPs proteins stratified according to the Child–Pugh score are given in Figure 3. The protein amounts decreased parallel to the disease progression, and the rank order of the enzymes was not markedly affected by the functional state of the liver. CYP2C9, CYP2E1, CYP1A2, and CYP3A4 showed the highest abundances, while CYP2B6, CYP1A1, and CYP2C19 were only found in traces (~1–2%) (Figure 3, Table S2).

The Jonckheere–Terpstra test evidenced a significant trend in the downregulation of CYP1A2 (*p* = 0.012), CYP2C9 (*p* = 0.029), CYP3A4 (*p* = 0.019), CYP2E1 (*p* = 0.004), UGT2B7 (*p* = 0.0001), and UGT2B15 (*p* = 0.013) protein abundances from class A to C classified livers.

### 2.3. Genotyping

Genotyping studies of *CYP2C19*, *CYP2D6*, and *CYP3A5* resulted in exclusion of samples genetically determined with the enzyme deficiency, i.e., *CYP2C19* – 2 controls, 1 HCV subject, and *CYP2D6* – 1 control, 3 HCV subjects. As for *CYP3A5* expression, 7 out of 58 HCV patients and 3 out of 20 control subjects were defined as expressers (*1/*3) with a protein abundance of 297.7 fmol/mg (±259.0). The *CYP3A5* non-expressers (*3/*3) protein levels were defined at 39.6 fmol/mg (±34.07).

## 3. Discussion

Information about the proteomic status of DMEs in the liver in its healthy and disease states provides insights into potential effects of liver pathologies on drug pharmacokinetics, and thus therapeutic responses as well as potential side effects. The correlation between DMEs mRNA and protein levels is not always satisfactory (in the present study it was especially affected by HCV infection). Therefore, reliable protein quantification information allows for better predictions than mRNA expression data. Our previous findings from studies including various liver pathologies (hepatic virus-induced liver damage, alcoholic liver disease, autoimmune hepatitis, primary biliary cholangitis, and primary sclerosing cholangitis) demonstrate a disconnection between the gene expression and protein abundance of DMEs [7].

So far, only limited information about the proteomic data of DMEs in relation to liver diseases has been published. The above-mentioned study [7], demonstrated that different liver pathologies affected various CYPs and UGTs. The HCV samples of the aforementioned study (21 samples are also included in the present analysis) demonstrated that the disease was associated with significant down-regulation of CYP2E1, CYP3A5, and UGT2B7 protein abundances (and unchanged levels of CYP1A1, CYP1A2, CYP2B6, CYP2C8, CYP2C9, CYP2C19, CYP2D6, CYP3A4, UGT1A1, UGT1A3, and UGT2B15). Those results mostly corroborate the findings of the present study, but analysis of a larger number of cases also revealed significant protein abundance reduction in CYP1A2, CYP2C19, CYP3A4, and UGT2B15, as well as a marked up-regulation of UGT1A1. These findings are supported by the proteomic (LC–MS/MS) analysis of Prasad et al. [9] for CYP2D6 (unchanged levels) as well as CYP1A2, CYP2E1, UGT2B7, and UGT2B15 (down-regulation) in 30 HCV liver specimens. The observed differences could be related to methodological specificities; however, they could also be related to analysis of liver tissues from patients with various stages of HCV liver disease (not defined in the study of Prasad et al. [9]).

The present study also provides as-of-yet unavailable information about the changes of DMEs in HCV livers in dependence on the organ functional stage (based on the Child–Pugh classification). Our previous study suggested that the stage of liver dysfunction (analyzed in the combined samples from five different liver pathologies) affected DMEs protein levels. In detail, the study revealed protein abundance down-regulation of CYP2E1 in class A livers, decreased levels of CYP2E1 and CYP2C8, CYP3A4, CYP3A5, and UGT2B7 in class B, as well as reduction in CYP2E1, CYP2C8, CYP3A4, CYP3A5, and CYP1A2, CYP2C9 in the class C tissues [7]. However, these findings, as stated above, are based on the merged results of five liver pathologies, with various contributions to each Child-–Pugh class. Thus, the advantage of the current study relies on the analysis of only one liver pathology, i.e., HCV. The results of the present study indicate that the Child–Pugh class A livers are characterized by significant up-regulation of UGT1A1, the class B livers by down-regulation of CYP2C19, CYP2E1, CYP3A4, UGT1A3, and UGT2B7, as well as the class C livers by significant reduction in CYP1A2 and UGT2B7.

The present analysis provides evidence that progression of liver dysfunction produced by HCV infection is associated with constant down-regulation of DMEs. The range of protein abundance changes were in most cases over two-fold, which suggested potential clinical relevance. The results of the present study can be used to explain pharmacokinetic changes observed in HCV infected patients [10]. This study demonstrated that CYP1A2, CYP2C19, CYP2D6, and CYP2E1 enzyme activity was differentially affected by the presence of liver disease. The activity of all enzymes decreased significantly, but the reduction depended on the organ functional state. However, Frye at al. did not specify underlying liver pathology, and stratified subjects according to the Child–Pugh classification into compensated liver disease (Child–Pugh score of 5) or decompensated liver disease (Child–Pugh score ≥ 6) [10]. Our quantitative proteomic data are in keeping with altered enzymatic activity in the liver disease patients, e.g., lower (40%) chlorzoxazone (CYP2E1 substrate) metabolic ratio in patients with moderate–severe liver disease, significantly (69%) lower caffeine (CYP1A2 substrate) metabolic ratio in decompensated liver disease (Pugh score ≥6), and no effects on the drug pharmacokinetics of the compensated disease (Pugh score =5) [10]. From this perspective, the current study findings are in keeping with clinical data on metabolic ratios of probe substrates for the studied enzymes (i.e., CYP1A2, CYP2C19, and CYP2E1) [10]. The CYP3A4 results of the present study are in line with clinical observations on midazolam (CYP3A4 substrate) pharmacokinetics reported by Pentikäinen, who demonstrated significantly higher oral bioavailability (38%) and 41% lower total clearance of the drug (and unchanged plasma protein binding and distribution) in patients with chronic liver disease (with undefined etiology) [11]. The pharmacokinetic study, which revealed unaltered pharmacokinetics of debrisoquine (substrate of CYP2D6) in mild or moderate (Child–Pugh classification) liver disease patients also corroborate our results. However, reduced urinary excretion of 4-hydroxydebrisoquine in patients with the liver disease was also observed [12].

The present study also demonstrated significantly elevated protein levels of UGT1A1 in Child–Pugh class A livers, and this observation is not in keeping with the study of Prasad et al. [9], who reported UGT1A1 to be below the lower limit of quantification in livers infected with HCV. However, our finding fits to the changes in the gene expression reported by Congiu et al. [13]. The latter study revealed higher levels of UGT1A1 in early stages of liver fibrosis; however, they noted down-regulation in more advanced stages of the disease. In the present study, mRNA UGT1A1 levels in the liver were similar in all Child–Pugh classes. Clinical pharmacokinetic study suggests that glucuronidation pathways are not affected by liver diseases (revised in [14]), which is also supported by the stable protein levels of UGT1A3 and UGT1A1 in the Child–Pugh class B and C HCV livers revealed in this study. However, in most studies reporting preservation of drug glucuronidation only patients with mild to moderate liver disease were recruited. More recent reports have revealed that some glucuronidation pathways could be down-regulated in more advanced stages of liver failure. Our study demonstrated lower levels of UGT2B7 and UGT2B15 in HCV affected livers, especially in the Child–Pugh class C livers. These findings may explain the reduced glucuronidation of morphine (UGT2B7) [15], lamotrigine (UGT1A4, UGT2B7) [16], zidovudine (UGT2B7) [17], mycophenolic acid (UGT1A9, UGT2B7) [18], or oxazepam (UGT2B15) [19] in patients with liver dysfunction. However, those studies might not identify the effects of the disease on individual UGT isoforms levels, since the substrate overlap of UGTs activity is frequently observed.

The protein abundance of DMEs in the control group from the present study are mostly in keeping with the results of other reports. It should be stated that the control liver source can (to some extent) affect results. The preliminary study compared DMEs protein abundances in organ donor livers and metastatic livers (used in studies as reference/control values) and demonstrated some differences [20]. The type of specimen analyzed, i.e., whole liver tissue or microsomal fraction applied for proteomic analysis [21], along with tissue preservation process [22] as well as methodological issues [23,24], can also produce some discrepancies. In the present study, the control group results of the most abundant CYPs (CYP2C9, CYP2E1, and CYP3A4) in metastatic livers free from pathological changes are in line with Vasilogianni et al.’s targeted proteomics results, but measured in microsomal fraction [25], and Couto et al., who measured CYPs in microsomal fraction using a global proteomic approach in the same type of control (metastatic liver) tissues [26].

In the current study, only Caucasian liver samples were included so that we could exclude the ethnic bias. In the literature the impact of ethnicity on drug pharmacokinetics was postulated, suggesting indirect differences in DMEs levels/activities [27]. Some of the inter-ethnic differences can be ascribed to genetic polymorphisms, which affect protein levels or enzymatic activity, e.g., higher frequencies of CYP3A5 allele expression (CYP3A5*3, CYP3A5*6, and CYP3A5*7) in African Americans compared to individuals of European, Native American, and Asian ancestry (affecting pharmacokinetics of tacrolimus) [28] or higher number of the slow metabolizer of CYP2B6 516 G > T allele in Africans Americans (46.7%) and Sub-Saharan Africans (45%) compared to Asians (17.4%), Hispanics (17.4%), Japanese (18%), and Caucasians (21.4%) (affecting efavirenz or atazanavir pharmacokinetics) [29,30]. In the present study 7 out of 58 HCV patients and 3 out of 20 control subjects were defined as expressers of CYP3A5 (*1/*3 genotype) with protein abundance levels of 297.7 fmol/mg (±259.0), in comparison to 39.6 fmol/mg (±34.07) in non-expressers (*3/*3 genotype). However, there is a paucity of expression and protein abundance information about DMEs in different ethnic groups. The available data suggest that ethnic origin does not have a substantial impact on DMEs levels [31]. It seems that nongenetic factors such as diet, weight, and environmental factors should be also highlighted as potential sources of inter-individual variation in drug pharmacokinetics. There is also evidence of age-dependent changes in the expression levels, protein abundances, and activities of DMEs [32,33]. However, our study groups are age-similar, and the impact of age on the results can be disregarded.

The changes in the DMEs expression/abundance can be, in part, ascribed to altered cytokine status produced by HCV infection in the liver. Hepatitis C is an inflammatory disease associated with elevated expression levels of IL(interleukin)-6 and TNF(tumor necrosis factor)-α [34]. It is also documented that liver-infiltrating T cells from chronic hepatitis C patients produced IFN(interferon)-γ [35], apoptotic hepatocytes released IL-1α [36], and macrophages exposed to HCV secreted IL-1β and IL-18 [37]. These cytokines could be involved in the transcriptional regulation of some DMEs and may explain the expression/abundance changes observed in the present study. Human hepatocyte culture experiments demonstrated that IL-6 exposure resulted in the down-regulation of genes coding for CYP3A4 and CYP2B6, as well as up-regulation of CYP1A2. This study did not evidence any impact of IL-1β, and no synergism between IL-6 and IL-1β on the CYPs genes expression [38]. The exposure of HepaRG cells to IL-6 produced the suppression of CYP1A2, CYP2B6, and CYP3A4 mRNA levels. Similar findings on CYP1A2, CYP2B6, and CYP3A4 mRNA expressions were observed in primary hepatocytes [39]. However, no suppression of CYP1A2, CYP2B6, and CYP3A4 mRNAs after exposure to IL-18 and IL-1β was observed [38,39]. The TNF-α suppressed expression of cyp1a1 gene in the hepatocyte cell line Hepa1c1c7 [40], and IFN-γ induced down-regulation of CYP1A2 and CYP3A4 expression in human hepatocytes [41]. The down-regulation of transcription factors such as hepatocyte nuclear factors (HNFs), NF(nuclear factor)-κB, along with several nuclear receptors such as pregnane X receptor (PXR) and constitutive androgen receptor (CAR), have been proposed to be responsible for suppression of the CYPs expression by inflammatory stimuli [40,42,43]. It was shown that PXR was involved in the IL-6-mediated down-regulation of CYP3A4 in HepG2 cells [44], and IL-1 mediated regulation of CYP3A4, with possible contribution of HNF(hepatocyte nuclear factor)4 [45]. An involvement of NF-κB in the down-regulation of CYP1A1/1A2 expression in hepatocytes was also reported [40]. Furthermore, PXR and CAR could also regulate phase II enzymes in hepatocytes [46]. However, not all results of the present study are in keeping with in vitro and ex vivo experimental findings; contrary results have also emerged from those reports, most likely due to different cell models, culture conditions, or experimental protocols.

The present study results can be also used to better scale PBPK models of DAAs, as there is missing information about pharmacokinetics in HCV patients with advanced liver disease (especially in Child–Pugh class C subjects). The study findings are in keeping with the available pharmacokinetic data and recommendations specified in the summaries of product information of DAAs. The significantly down-regulated levels of CYP1A2 (and CYP3A4 and UGT1A1) could explain the altered pharmacokinetics of pibrentasvir, whose area under the concentration–time curve (AUC) differed by 26% or less in patients with Child–Pugh class A or B cirrhosis and increased to 2.1-fold for those with class C [47].

The protein levels of CYP2B6 were not affected by the stage of liver failure. Velpatasvir is metabolized via this enzymatic pathway (also via CYP2C8 and CYP3A4), and its pharmacokinetics was not affected by liver dysfunction, which supports the proteomic findings of the present study. The drug AUC was comparable in non-HCV Child–Pugh classes B and C patients with normal hepatic function subjects [48].

The CYP2C8 protein abundance, similar to CYP2B6, was not altered in the samples from HCV-infected patients. The enzyme contributes to dasabuvir (and as stated above of velpatasvir) metabolism. The AUC values of dasabuvir were similar in healthy subjects and the Child–Pugh class A patients. However, in the Child–Pugh class B patients a 16% AUC reduction of the drug was observed, which was paralleled by a 57% decrease in M1 (major metabolite) AUC values. The class C subjects were characterized by elevated AUCs for dasabuvir (325%) and M1 (77%) [49]. According to the summary of product characteristics, dasabuvir should not be administered to the Child–Pugh class B and C patients. The down-regulation in CYP2C8 protein levels in the liver could contribute to the observed changes in the drug pharmacokinetics.

The pharmacokinetic information about simeprevir, a CYP2C19 substrate (also a substrate of CYP3A4 and CYP2C8) in liver dysfunction patients is not equivocal. Sekar et al. [50] observed equal AUC in non-HCV Child–Pugh class A and B subjects. Other trials reported two-fold higher AUC in non-HCV Child–Pugh class B and C patients [51] or 2.4- and 5.2-fold AUC increases in the Child–Pugh class B and C patients, respectively, compared to healthy individuals. However, contribution of CYP3A4 down-regulation to the observed changes cannot be excluded. Therefore, simeprevir should not be used in Child–Pugh class C patients and caution should be taken with Child–Pugh class B subjects, as stated in the manufacturer recommendations [52]. The reported alterations in simeprevir pharmacokinetic characteristics can be considered to be in keeping with the protein abundance changes observed in the present study, since the down-regulation of CYP2C19 protein abundance was found, significant from the Child–Pugh class B.

The documentation in the present study of significant down-regulation of CYP3A4 levels are in line with pharmacokinetic studies and recommendations for the clinical use of elbasvir/grazoprevir, glecaprevir/pibrentasvir, and sofosbuvir/velpatasvir/voxilaprevir (all agents, except for sofosbuvir, are substrates of CYP3A4). Clinical guidelines mark those combined medications as not recommended or contraindicated in the Child–Pugh class B and C patients (also due to unavailability of the relevant data). Results of pharmacokinetic studies are in part inconsistent (due to complexity of factors affecting drug kinetics in liver failure patients) with the present study findings; however, some could indicate reduced CYP3A4 metabolic capacity of the liver, i.e., increased steady-state exposure and C_max_ of grazoprevir changing with the Child–Pugh class [53] or pibrentasvir (also a substrate for CYP1A2 down-regulated in the present study) AUC increase by 51%, 31%, and 5.2-fold in patients with the Child–Pugh A, B, and C, respectively [47]. Other substrates of CYP3A4, i.e., daclatasvir or elbasvir are highly protein bound (>99%) and characterized by a low hepatic extraction ratio. Therefore, liver function deterioration could not influence the unbound fraction of daclatasvir in the Child–Pugh class B and C patients, in comparison with HCV-infected controls [3]. Likewise, elbasvir exposure was comparable in HCV patients with Child–Pugh class B liver cirrhosis and healthy controls [54].

In addition to disease-related changes in the protein abundance of distinct CYP enzymes as investigated in our study, the applied pharmacotherapy in HCV patients may be affected by the inhibitory potential of several DAAs, i.e., CYP1A2—glecaprevir, pibrentasvir; CYP3A4/5—asunaprevir, daclatasvir, dasabuvir, elbasvir, glecaprevir, grazoprevir, paritaprevir, pibrentasvir, simeprevir, velpatasvir; and UGT1A1—glecaprevir, pibrentasvir [3,4]. This information is of importance as some of these agents are available as fixed-dose combinations, e.g., elbasvir/grazoprevir or glecaprevir/pibrentasvir. This information, apart from CYPs protein abundance levels, should be implemented in the construction of PBPK models.

## 4. Materials and Methods

### 4.1. Liver Samples

The control samples were harvested from metastatic livers, from a site at least 5 cm distance of the tumor site. The tissues were collected from Caucasian patients, aged 63 ± 10 years, 11 males and 9 females, diagnosed with metastatic colon cancer. The collected tissues did not show any pathological signs as confirmed by histological examination (the samples were used as the controls in the previously published study [7]).

HCV (diagnosed according to the standard clinical criteria) liver parenchymal tissue samples were dissected from the patients requiring liver transplantation. The liver tissue specimens were harvested during elective liver transplantation from the organ immediately after excision. The stage of liver dysfunction was classified according to the Child–Pugh score. Characteristics of the subjects are presented in Table 2. The whole medication information is available for the control samples, i.e., one patient was treated with bisoprolol, furosemide, and tamsulosin (hypertension and prostate hypertrophy), one was treated with bepridil (hypertension), and another one was medicated with amlodipine (hypertension). None of these drugs are known to be a potent regulator of CYP or UGT enzymes. The HCV liver samples were collected in the years 2007–2019, and treatment standards for HCV were modified several times in this period, which is a limitation of the samples. We were only able to select samples without co-existing co-morbidities.

Tissue biopsies were taken from livers (control and pathological) under standard general anesthesia not later than 15 min after blood flow arrest. The liver samples were immediately snap-frozen in liquid nitrogen for protein analysis or immersed in RNAlater (Applied Biosystems, Darmstadt, Germany) for RNA analysis, and then stored at −80 °C. The study protocol was approved by the Bioethics Committee of the Pomeranian Medical University.

### 4.2. mRNA Isolation and Quantitative Real-Time RT-PCR

Total RNA was isolated from 25 mg of each tissue sample using a Direct-zol RNA MiniPrep kit (Zymo Research, Irvine, CA, USA). RNA concentration and purity was assessed using a DS-11 FX spectrophotometer (Denovix, Wilmington, DE, USA). cDNA was prepared using a SuperScript^®^ VILO™ cDNA Synthesis Kit (Thermo Fisher Scientific, Waltham, MA, USA), with 500 ng of total RNA for 20 µL of reaction volume, according to the manufacturer’s procedure. The gene expression levels were examined in duplicate using TaqMan Fast Advanced Master Mix and pre-validated TaqMan assays: CYP1A1 (Hs00153120_m1), CYP1A2 (Hs00167927_m1), CYP2B6 (Hs03044631_m1), CYP2C8 (Hs02383390_s1), CYP2C9 (Hs02383631_s1), CYP2C19 (Hs00426380_m1), CYP2D6 (Hs00164385_m1), CYP2E1 (Hs00559367_m1), CYP3A4 (Hs00604506_m1), CYP3A5 (Hs01070905_m1), UGT1A1 (Hs02511055_s1), UGT1A3 (Hs04194492_g1), UGT2B7 (Hs00426592_m1), UGT2B15 (Hs00870076_s1) in ViiA 7 Real-Time PCR System (Life Technologies, Waltham, MA, USA). Threshold values for each gene were set manually and mean C_T_ (cycles of threshold) values were recorded. Relative mRNA expression was calculated by the 2^−ΔCt^ method, which was normalized to the mean expression value obtained for the housekeeping genes: GAPDH (Hs99999905_m1), HMBS (Hs00609297_m1), PPIA (Hs04194521_s1), RPLP0 (Hs99999902_m1), RPS9 (Hs02339424_g), and by 2^−ΔΔCt^ method, which was additionally normalized to the mean value for the control group.

### 4.3. Genotyping

Genomic DNA was extracted from tissue samples using a Tissue DNA Purification Kit (EURx, Gdansk, Poland) and subsequently standardized to a uniform concentration (20 ng/μL) before being stored at −20 °C. All samples were genotyped for common lack-of-function variants affecting protein concentration (i.e., stop-codons, frameshifts, and splicing defects) using ViiA7 Fast Real-Time PCR System and pre-validated TaqMan assays (Life Technologies, Carlsbad, CA, USA). The following variants were evaluated: CYP2C19*2 (rs4244285, Assay ID: C__25986767_70), CYP2D6*3 (rs35742686, C__32407232_50), CYP2D6*4 (rs3892097, C__27102431_D0), and CYP3A5*3 (rs776746, C__26201809_30). Additionally, CYP2D6 gene deletion (CYP2D6*5) was evaluated using the qPCR method with TaqMan probes for CYP2D6 (Hs00010001_cn) and reference RPPH1 gene.

### 4.4. Protein Quantification by LC−MC/MS

Tissues placed in liquid nitrogen were mechanically disrupted in a stainless-steel mortar system. Approximately 40 mg of tissue powder of each sample was lysed with 1 mL of 0.2% SDS and 5 mM of EDTA containing 5 µL/mL of Protease Inhibitor Cocktail Set III (Merck, Darmstadt, Germany) for 30 min at 4 °C on a platform shaker with 40 rpm (Polymax 1040, Heidolph, Schwabach, Germany). Total protein content of the whole tissue lysates was determined by bicinchinonic acid assay (BCA, Thermo Fisher) and 100 µg of each sample was processed using a filter aided sample preparation (FASP) [55]. Protein quantification of nine CYP (CYP1A2, CYP2B6, CYP2C8, CYP2C9, CYP2C19, CYP2D6, CYP2E1, CYP3A4, and CYP3A5) and four UGT (UGT1A1, UGT1A3, UGT2B7, and UGT2B15) enzymes were measured by mass spectrometry-based targeted proteomics using the validated LC−MS/MS method [56]. With the exception of UGT1A3, an additional proteospecific peptide was analyzed for each protein in the same manner as the 13 validated peptides (i.e., 2 proteospecific peptides have been used for each enzyme). One peptide was used for quantification whereas the other served as a qualifier for the presence of the specific protein. For all peptides and their isotope-labeled internal standard peptides, three mass transitions were used, respectively. The calculated protein values represent the mean of at least 2–3 mass transitions/peptide. The final protein abundance for each enzyme was normalized to the individual mass of tissue used in the tryptic digest (fmol/mg).

### 4.5. Statistical Analysis

The mRNA and protein expression data were means ± standard deviation and coefficient of variation %. The median as well as minimum and maximum values are given in Tables S1 and S2 of Supplementary Information. Differences between the study groups were evaluated using the nonparametric Kruskal–Wallis test for multiple comparisons with the post hoc Dunn’s test, and correlations with the Spearman rank test. The Jonckheere–Terpstra test for ordered differences was used to determine the significance of a trend in protein abundance along the liver functional state (using the Child–Pugh classification). The *p* values of <0.05 were considered significant. The statistical calculations were performed using Statistica 13.3 Software Package (TIBCO Software Inc., Palo Alto, CA, USA).

## 5. Conclusions

In conclusion, it can be stated that the study provides information about the proteomic data of clinically relevant DMEs in hepatitis C-infected livers, also in relationship to the disease stage classified according to the Child–Pugh score. The disease significantly down-regulated the protein abundance of CYP1A2, CYP2C19, CYP2E1, CYP3A4, UGT2B7, and UGT2B15. The levels of CYP1A1, CYP2B6, CYP2C8, CYP2C9, CYP2D6, as well as UGT1A3, remained stable, whereas the protein amount of UGT1A1 was up-regulated. DMEs down-regulation mostly developed in the Child–Pugh class B (only CYP1A1 started to be decreased in the class C). The rank order of the enzymes was not markedly affected by the liver functional states, i.e., CYP2C9, CYP2E1, CYP1A1, and CYP3A4 showed the highest abundances, while CYP2B6, CYP1A1, and CYP2C19 were found in trace amounts (~1–2%). These findings indicate that the HCV liver has preserved capacity of drug metabolism in the Child–Pugh class A stage. The results from the present study can be incorporated into PBPK models in order to get more precise predictions of drug pharmacokinetics or drug–drug interactions and thus appropriate drug dose-adjustments in patients with HCV liver dysfunction. Refinement of the existing models can be of special importance for drugs where their clinical application is currently limited to mild stages of HCV liver disease (e.g., prescription of ombitasvir/paritaprevir/ritonavir or dasabuvir is restricted to the Child–Pugh score A, since the efficacy and safety of these agents were not studied in the Child–Pugh score B and C patients). This approach can open, based on more adequate estimations, clinical studies on drugs not registered for application in patients with advanced liver diseases since, for ethical reasons and risks, such trials without sufficient entry data are not implemented. The results of the present study can be combined with the findings on drug transporters protein abundances in the same set of HCV patients [8]. The combined picture of DMEs and transporters in the pathological livers can contribute to building PBPK models in HCV patients.

## Figures and Tables

**Table 1:** Correlations (Spearman coefficient, r) between protein abundance and mRNA expression levels of CYPs and UGTs in HCV livers (HCV), HCV liver disease stages (Child–Pugh class A, B and C), as well as the controls.

mRNA vs. Protein Correlation Coefficient					
Protein	Controls *n* = 20 ^a^	HCV *n* = 58 ^b^	Child–Pugh Class A *n* = 30 ^c^	Child–Pugh Class B *n* = 21 ^d^	Child–Pugh Class C *n* = 7 ^e^
CYP1A1	0.664 ***	0.584 ***	0.638 ***	0.505 *	0.414
CYP1A2	0.824 ***	0.652 ***	0.517 **	0.642 **	0.571
CYP2B6	0.612 **	0.332 **	0.426 *	−0.003	0.786 *
CYP2C8	0.645 **	0.025	−0.078	−0.177	0.571
CYP2C9	0.620 **	0.202	0.238	−0.203	0.679
CYP2C19	0.325	−0.044	−0.010	−0.180	0.715
CYP2D6	0.586 **	0.357 **	0.468 *	0.389	−0.321
CYP2E1	0.352	0.118	0.321	−0.091	0.000
CYP3A4	0.889 ***	0.466 ***	0.400 *	0.348	0.714
UGT1A1	0.675 **	0.280 *	0.378 *	0.073	0.536
UGT1A3	0.699 ***	0.306 *	0.092	0.348	0.857 *
UGT2B7	0.800 ***	0.235	0.051	0.094	−0.214
UGT2B15	0.725 ***	0.317 *	0.295	−0.019	0.679

* *p* < 0.05, ** *p* < 0.01, *** *p* < 0.001; ^a^ CYP2C19 *n* = 18, CYP2D6 *n* = 19; ^b^ CYP2C19 *n* = 57, CYP2D6 *n* = 55; ^c^ CYP2D6 *n* = 29; ^d^ CYP2D6 *n* = 19; ^e^ CYP2C19 *n* = 6.

**Table 2:** Characteristics of the subjects (mean ± SD).

Parameter/Disease	Control *n* = 20	HCV *n* = 58	Ch-P A *n* = 30	Ch-P B *n* = 21	Ch-P C *n* = 7
Sex [male/female]	11/9	30/28	16/14	11/10	3/4
Age [years]	63 ± 10	56 ± 7	57 ± 7	55 ± 8	52 ± 9
Total bilirubin [mg/dL]	0.59 ± 0.25	1.75 ± 1.26	1.03 ± 0.57	2.05 ± 0.84	3.62 ± 1.78
Albumin [g/dL]	3.89 ± 0.38	3.38 ± 0.57	3.67 ± 0.49	3.23 ± 0.45	2.71 ± 0.40
INR	1.14 ± 0.21	1.30 ± 0.28	1.20 ± 0.22	1.29 ± 0.17	1.71 ± 0.36

HCV—hepatitis C; Ch—P: A, B, C—Child–Pugh Class A, B, C; INR—International Normalized Ratio.

## Data Availability

Not applicable.

## Supplementary Materials

The supporting information can be downloaded at: https://www.mdpi.com/article/10.3390/ijms24054543/s1.
