# Supplementary material for: Protein Abundance of Drug Metabolizing Enzymes in Human Hepatitis C Livers
Source: Int J Mol Sci. 2023 Feb 25;24(5):4543. doi: 10.3390/ijms24054543 (PMC10002520; doi:10.3390/ijms24054543)
Supplement: Supplementary file 1 [file ijms-24-04543-s001.zip › ijms-2146685-supplementary.pdf]

Protein abundance of drug metabolizing enzymes in human hepatitis C livers

Marek Drozdzik, Joanna Lapczuk-Romanska, Christoph Wenzel, Lukasz Skalski, Sylwia Szelag-Pieniek, Mariola Post, Arkadiusz Parus, Marta Syczewska, Mateusz Kurzawski, Stefan Oswald

Supplemental Table S1. mRNA expression data of CYPs and UGTs in HCV cirrhotic livers (HCV (A+B+C)) and disease stages (Child–Pugh class A, B and C) as well as in the control

	median	min	max	mean	SD	CV %
CYP1A1						
Ch-P A	0.964	0.158	4.110	1.240	1.011	82
Ch-P B	0.823	0.048	4.579	1.357	1.454	107
Ch-P C	0.265	0.106	1.696	0.687	0.698	102
HCV (A+B+C)	0.878	0.048	4.579	1.215	1.164	96
Control	0.385	0.150	4.937	1.000	1.245	117
CYP1A2						
Ch-P A	0.364	0.015	1.271	0.491	0.357	73
Ch-P B	0.300	0.037	1.684	0.444	0.446	100
Ch-P C	0.143	0.004	1.282	0.302	0.462	153
HCV (A+B+C)	0.310	0.004	1.684	0.451	0.401	89
Control	0.846	0.179	3.001	1.000	0.680	68
CYP2B6						
Ch-P A	0.925	0.131	4.242	1.161	0.931	80
Ch-P B	0.914	0.296	2.057	1.029	0.468	45
Ch-P C	0.442	0.150	4.361	1.107	1.488	134
HCV (A+B+C)	0.911	0.131	4.361	1.107	0.869	79
Control	0.952	0.075	2.895	1.000	0.647	65
CYP2C8						
Ch-P A	0.616	0.285	1.263	0.657	0.268	41
Ch-P B	0.583	0.281	1.401	0.680	0.334	49
Ch-P C	0.371	0.082	1.240	0.542	0.399	74
HCV (A+B+C)	0.586	0.082	1.401	0.651	0.307	47
Control	1.013	0.280	2.242	1.000	0.454	45
CYP2C9						
Ch-P A	1.562	0.577	3.710	1.747	0.669	38
Ch-P B	1.355	0.658	2.969	1.408	0.508	36
Ch-P C	1.083	0.122	1.529	0.906	0.568	63
HCV (A+B+C)	1.431	0.122	3.710	1.523	0.656	43
Control	1.021	0.275	1.491	1.000	0.313	31
CYP2C19						
Ch-P A	0.273	0.103	0.600	0.278	0.122	44
Ch-P B	0.247	0.110	0.698	0.303	0.147	49
Ch-P C	0.311	0.213	1.259	0.528	0.430	81
HCV (A+B+C)	0.266	0.103	1.259	0.314	0.194	62
Control	0.757	0.153	2.361	1.000	0.775	78
CYP2D6						
Ch-P A	0.621	0.028	1.694	0.735	0.393	53

Ch-P B	0.564	0.180	3.196	0.922	0.875	95
Ch-P C	0.588	0.290	1.281	0.691	0.333	48
HCV (A+B+C)	0.620	0.028	3.196	0.794	0.597	75
Control	0.898	0.294	1.983	1.000	0.485	49
CYP2E1						
Ch-P A	0.568	0.061	1.046	0.545	0.255	47
Ch-P B	0.508	0.088	0.852	0.501	0.230	46
Ch-P C	0.454	0.092	0.664	0.395	0.242	61
HCV (A+B+C)	0.523	0.061	1.046	0.511	0.245	48
Control	1.013	0.328	1.556	1.000	0.369	37
CYP3A4						
Ch-P A	0.490	0.075	1.498	0.594	0.411	69
Ch-P B	0.390	0.049	1.780	0.528	0.493	93
Ch-P C	0.951	0.002	2.559	0.900	0.934	104
HCV (A+B+C)	0.430	0.002	2.559	0.607	0.526	87
Control	0.870	0.068	2.454	1.000	0.701	70
UGT1A1						
Ch-P A	0.993	0.181	1.900	1.018	0.492	48
Ch-P B	0.891	0.373	3.040	1.018	0.623	61
Ch-P C	1.085	0.304	4.397	1.352	1.398	103
HCV (A+B+C)	0.993	0.181	4.397	1.058	0.690	65
Control	0.860	0.274	2.668	1.000	0.591	59
UGT1A3						
Ch-P A	0.263	0.038	1.055	0.367	0.251	68
Ch-P B	0.431	0.168	1.051	0.441	0.242	55
Ch-P C	0.241	0.125	0.890	0.362	0.282	78
HCV (A+B+C)	0.327	0.038	1.055	0.393	0.250	63
Control	0.808	0.298	2.474	1.000	0.602	60
UGT2B7						
Ch-P A	0.521	0.226	1.132	0.609	0.248	41
Ch-P B	0.526	0.047	1.020	0.498	0.273	55
Ch-P C	0.235	0.028	1.301	0.367	0.429	117
HCV (A+B+C)	0.501	0.028	1.301	0.539	0.289	54
Control	0.925	0.398	1.943	1.000	0.409	41
UGT2B15						
Ch-P A	1.677	0.427	4.326	1.858	0.852	46
Ch-P B	1.114	0.577	5.389	1.688	1.275	76
Ch-P C	1.046	0.044	1.693	0.961	0.593	62
HCV (A+B+C)	1.326	0.044	5.389	1.688	1.028	61
Control	0.884	0.330	2.813	1.000	0.549	55

Relative quantity of each transcript is presented, normalized to the mean value for the control group ($2^{-\Delta\Delta CT}$ method); CV% - coefficient of variation; min-minimum; max-maximum; SD- standard deviation

Supplemental Table S2. Protein abundance (fmol/mg) of the P450s and UGTs in HCV cirrhotic livers (HCV (A+B+C)) and disease stages (Child–Pugh class A, B and C) as well as in the control.

**Protein** [fmol/mg]	**median**	**min**	**max**	**mean**	**SD**	**CV %**	**positive samples**
**CYP1A1**							
Ch-P A	38.92	0.00	172.72	50.99	44.72	88	27/30
Ch-P B	34.08	0.00	96.52	34.13	24.51	72	18/21
Ch-P C	11.59	0.00	95.98	25.77	34.02	132	5/7
HCV (A+B+C)	34.10	0.00	172.72	41.84	38.05	91	50/58
Control	21.50	0.00	190.27	37.68	50.75	135	18/20
**CYP1A2**							
Ch-P A	417.56	54.81	2579.01	687.47	653.63	95	30/30
Ch-P B	235.94	8.80	2000.00	530.71	584.24	110	21/21
Ch-P C	58.84	5.70	3019.78	495.06	1115.22	225	7/7
HCV (A+B+C)	315.49	5.70	3019.78	607.49	689.31	113	58/58
Control	768.55	40.58	2645.59	953.10	738.45	77	20/20
**CYP2B6**							
Ch-P A	98.80	14.42	714.38	122.38	123.73	101	30/30
Ch-P B	108.82	11.31	327.99	106.15	66.84	63	21/21
Ch-P C	91.81	0.00	253.54	136.23	110.86	81	6/7
HCV (A+B+C)	102.89	0.00	714.38	118.18	103.69	88	57/58
Control	91.57	14.28	292.68	103.05	70.91	69	20/20
**CYP2C8**							
Ch-P A	441.15	79.33	1822.19	556.65	402.90	72	30/30
Ch-P B	324.49	27.64	1282.94	383.07	265.64	69	21/21
Ch-P C	438.45	17.58	1061.03	414.16	362.21	87	7/7
HCV (A+B+C)	431.21	17.58	1822.19	476.61	358.10	75	58/58
Control	532.83	101.21	1136.74	538.74	309.09	57	20/20
**CYP2C9**							
Ch-P A	2127.44	117.79	5517.80	2271.05	1302.05	57	30/30
Ch-P B	1849.97	55.28	5358.50	1792.89	1234.03	69	21/21
Ch-P C	1189.99	56.05	3599.48	1287.94	1200.44	93	7/7
HCV (A+B+C)	1919.72	55.28	5517.80	1979.27	1290.29	65	58/58
Control	2007.57	630.81	5676.82	2290.74	1276.02	56	20/20
**CYP2C19**							
Ch-P A	97.07	0.00	331.75	82.46	74.53	90	22/30
Ch-P B	43.22	0.00	241.19	58.29	68.10	117	13/21
Ch-P C	50.65	0.00	93.11	48.71	34.88	67	5/6
HCV (A+B+C)	69.48	0.00	331.75	70.00	69.36	99	40/57
Control	142.26	0.00	484.49	158.84	130.06	86	15/18
**CYP2D6**							
Ch-P A	267.43	43.44	620.00	287.80	152.77	53	29/29
Ch-P B	287.90	33.09	987.34	325.36	251.75	77	19/19
Ch-P C	164.24	37.53	507.12	204.60	160.91	79	7/7
HCV (A+B+C)	258.87	33.09	987.34	290.19	193.63	67	55/55
Control	292.45	55.71	852.82	364.67	239.57	66	19/19

**CYP2E1**							
Ch-P A	1309.31	102.88	4681.23	1556.95	1061.46	68	30/30
Ch-P B	587.85	36.86	2387.59	841.79	593.38	70	21/21
Ch-P C	513.47	122.55	3413.52	1190.05	1329.37	112	7/7
HCV (A+B+C)	1055.84	36.86	4681.23	1253.73	997.10	80	58/58
Control	1394.06	272.07	3407.44	1549.54	814.53	53	20/20
**CYP3A4**							
Ch-P A	456.10	37.50	1937.67	588.81	452.50	77	30/30
Ch-P B	193.56	7.54	1126.37	323.88	315.60	97	21/21
Ch-P C	89.72	0.00	1421.24	612.49	705.33	115	6/7
HCV (A+B+C)	336.19	0.00	1937.67	495.75	456.73	92	57/58
Control	672.99	99.21	2634.15	967.18	745.25	77	20/20
**CYP3A5**							
Ch-P A	34.89	14.98	388.78	69.85	93.83	134	30/30
Ch-P B	38.74	0.00	228.86	53.44	57.41	107	59.41885
Ch-P C	30.91	15.68	83.09	39.16	26.22	67	7/7
HCV (A+B+C)	34.89	0.00	388.78	60.20	76.34	127	57/58
Control	50.66	20.96	679.89	108.82	157.78	145	20/20
**UGT1A1**							
Ch-P A	1088.71	351.92	2666.97	1193.62	617.95	52	30/30
Ch-P B	1096.75	95.74	2283.39	1045.42	542.46	52	21/21
Ch-P C	771.96	496.80	2705.02	1157.68	824.09	71	7/7
HCV (A+B+C)	1037.17	95.74	2705.02	1135.62	611.43	54	58/58
Control	619.52	182.88	1123.72	641.33	309.24	48	20/20
**UGT1A3**							
Ch-P A	173.09	22.14	723.94	201.59	164.01	81	30/30
Ch-P B	123.01	30.98	449.01	163.90	109.10	67	21/21
Ch-P C	132.28	73.17	329.48	144.44	88.98	62	7/7
HCV (A+B+C)	152.54	22.14	723.94	181.04	138.53	77	58/58
Control	205.20	80.90	657.50	235.37	151.87	65	20/20
**UGT2B7**							
Ch-P A	1569.70	318.02	4663.00	1739.64	1002.68	58	30/30
Ch-P B	1003.76	0.00	3642.30	1059.29	746.98	71	20/21
Ch-P C	397.70	0.00	734.09	390.52	240.72	62	6/7
HCV (A+B+C)	1039.07	0.00	4663.00	1330.48	968.04	73	56/58
Control	1700.99	344.22	3455.79	1904.59	913.12	48	20/20
**UGT2B15**							
Ch-P A	563.55	127.38	1314.02	540.11	288.00	53	30/30
Ch-P B	360.11	139.75	1637.92	427.85	326.19	76	21/21
Ch-P C	218.37	101.54	2245.26	516.23	767.31	149	7/7
HCV (A+B+C)	392.91	101.54	2245.26	496.58	379.86	76	58/58
Control	550.30	174.36	1670.44	629.15	346.94	55	20/20

All results are given in fmol/mg of the analyzed tissue. CV% - coefficient of variation; min-minimum; max-maximum; SD- standard deviation
